# The mental health, quality of life and life satisfaction of internally displaced persons living in Nakuru County, Kenya: a critique

**DOI:** 10.1186/s12889-021-11176-y

**Published:** 2021-06-22

**Authors:** Robson Mandishekwa

**Affiliations:** Department of Economics, Midlands State University, Gweru, Zimbabwe

**Keywords:** Satisfaction with life, Internally displaced persons, Temporal satisfaction with life scale

## Abstract

The study titled ‘[t] he mental health, quality of life and life satisfaction of internally displaced persons living in Nakuru County, Kenya’ by Getanda, Papadopoulos and Evans identifies a critical area and contributed significantly to literature. Despite the contribution, there are some issues in that study that deserve attention. For example, the use of the satisfaction with life scale instead of the temporal satisfaction with life scale, for a pre-and post-displacement study, is questionable. It is important to note that the scores from the satisfaction with life scale can only measure life satisfaction at a particular point while the temporal satisfaction with life can be used to determine life satisfaction between pre-and post-displacement periods. Again, conflicting statements in the abstract and methods sections have been noted, where the abstract refers to refugee camps while methods refer to internally displaced persons’ camps. Finally, some reported statistics have been found to have errors. It is important to highlight these critical issues to readers for a better understanding. This study, therefore, endeavours to critique that study and clarify some of these issues so that readers get better understandings. Recommendations for future studies are made.

## Background

Getanda, Papadopoulos and Evans [1] identified a critical under-researched area, that is, satisfaction with life (SWL) among internally displaced persons. The importance of SWL, in general, has been widely acknowledged by several authors like Diener, Sapyta and Suh [2], Anielski [3], Brey [4] and Ferrer-i-Carbonell [5]. One such importance is that SWL impacts positively on productivity (Ivlevs, [6]; Arora & Rada, [7]). Thus, SWL contributes to the economic development of a nation because a happy society tends to be more productive (Ivlevs, [6]). However, studies on SWL among the displaced population remain scarce; therefore, Getanda, Papadopoulos and Evans [1] contributed in that area. However, anomalies in such pioneer studies may lead to misunderstandings. Therefore, this study clarifies areas with potential misunderstandings in the study by Getanda, Papadopoulos and Evans [1].

Although SWL is very important, its measurement has been widely debated with some authors preferring single-item scales while others favour multi-item scales. Among the multi-item scales, the satisfaction with life scale (SWLS) by Diener, Emmons, Larsen and Griffin [8] has been widely used. The current study, therefore, shows that while the SWLS is widely used, its application to measure changes in SWL between pre-and post-displacement periods is questionable. This study argues that the most appropriate scale is the Temporal Satisfaction With Life Scale (TSWLS) by Pavot, Diener and Suh [9]) which is able to determine SWL for three periods; past, present and future.

Kenya has been ranked number seven in Africa in terms of hosting conflict-induced IDPs (Getanda, Papadopoulos and Evans [1]). With Africa hosting most IDPs, this makes Kenya one of the good case studies on forced migration. Therefore, a study on IDPs in Kenya, may be regarded as a good starting point in such studies.

## Main text

Despite the importance of SWL, studies on SWL among IDPs remain scarce. Among the first authors to study SWL in IDPs are Getanda, Papadopoulos and Evans [1]. The study by Getanda, Papadopoulos and Evans [1] has made significant contributions to existing literature on SWL among IDPs. In Africa, conflict has led to displacements (IDMC, [10]) thereby possibly reducing SWL as found by the three authors. Also, Africa hosts a significant number of IDPs in the world (Norwegian Refugee Council, [11]) with Kenya being the host to the majority of IDPs (Verwimp and Maystadt [12]) thereby justifying the choice of the case study area. Also, the study findings were obtained in line with good research practices as reported.

However, there are three issues that deserve attention. The first concerns the use of the SWLS. Using the SWLS and concluding that SWL has fallen after displacement may be wrong because the SWLS scores measure only current SWL. Thus, association between displacement and SWL cannot clearly be shown. In such studies, it would have been more proper to use the TSWLS. The TSWLS enables one to compare previous, current and future SWL (Pavot, Diener and Suh [9]). While the results of the study may be plausible, they could have been improved by using the TSWLS to determine whether there is any association between displacement and SWL. Thus, it may be possible that average SWL between pre- and post-displacement periods may not be statistically different thereby implying that SWL remained the same as suggested by the stoicism theory which predicts life as gently flowing (Drakulic, [13]) despite adversity (Sirgy, [14]). Comparative quantitative data may probably indicate this. While the qualitative data somehow reflects the difference, it may have been complemented by using the TSWLS which can show the association between displacement and SWL using appropriate statistical methods. Again, since the authors state that comparison studies do not exist, self-comparison between the two periods may have improved results interpretation by use of a case-crossover design using the TSWLS data.

The second issue concerns the terminology on IDPs. Although there are varying definitions of IDPs, the definition of IDPs implied in the study is at least questionable. There seem to be contradictions between definitions used in the methods and abstract sections. While the methods section states that data was gathered from IDP camps, the abstract refers to refugee camps. By so doing one can deduce that the authors defined IDPs as refugees. However, this contradicts existing definitions of IDPs including the one by IDMC [15] where IDPs are defined as people who have been forced to migrate but do not cross internationally recognised borders. Again, IDPs are “... persons who have been forced or obliged to flee or to leave their homes or places of habitual residence, ... who have not crossed an internationally recognised state border” (UN, [16] p.1). Additionally, Opukri and Ibaba [17] define IDPs as aliens in their own communities. These definitions concur that IDPs do not cross international borders hence are not the same as refugees. Thus, stating that data was collected from refugee camps results in data being questionable for an IDP study unless further explanations are given. The key question one may ask is: Are refugee camps the same as IDP camps? This wording on its own needs clarification to reconcile it with the rest of the study. Confusion may also arise from the study because Verwimp and Maystadt [12] acknowledged Kenya as a host to the majority of refugees in Africa. Being a host to most refugees means that once the word refugee is mentioned about Kenya, one is likely to understand the word in its ordinary sense.

The third issue is on results reported in Table 2 presented by Getanda, Papadopoulos and Evans [1]. The trio’s Table 2 shows that 57 study participants had children. Given 100 participants, this translates to 57%, but they reported it as 27%. This anomaly has the potential for questioning the authenticity and reliability of results. This may however be a typing error. The three issues discussed deserve attention for readers to get a better understanding of the study.

## Conclusions

The results found by Getanda, Papadopoulos and Evans [1] could have been improved by the use of the TSWL. The absence of comparison data for SWL between the pre-and post-displacement periods has important implications in studies involving stressful life events. Such comparisons will enable conclusions to be made on whether displacement reduces SWL. Therefore, policies to enhance SWL may be advocated once it is established that internal displacement leads to reduced SWL.

Understanding the difference between IDPs and refugees is critical. This may assist in understanding potential ways to support them. For example, while refugees are protected by international laws, IDPs are not. As such, problems with these classifications may lead to problems where those deserving support may be denied.

Taken together the identified errors, can lead to problems with otherwise a good research. Therefore, future studies need to avoid such errors.

## Data Availability

Not Applicable.

## Abbreviations

IDPs: Internally Displaced Persons
SWL: Satisfaction with life
TSWLS: Temporal Satisfaction With Life Scale

## References

[1] Getanda EM, Papadopoulos C, Evans H (2015). The mental health, quality of life and life satisfaction of internally displaced persons living in Nakuru County, Kenya. BMC Public Health.

[2] Diener E, Sapyta JJ, Suh EM (1998). Subjective well-being is essential to wellbeing. Psychol Inq.

[3] Anielski M (2007). The economics of happiness: building genuine wealth.

[4] Brey P. Well-Being in Philosophy, Psychology, and Economics. In: Brey PA, Briggle A, Spence E, editors. The good life in a technological age: Routledge; 2012. p. 15–34. (Pre-print version).

[5] Ferrer-i-Carbonell A (2013). Happiness economics. Ser J Span Econ Assoc.

[6] Ivlevs A (2015). Happy moves? Assessing the link between satisfaction and emigration intentions, IZA DP No. 9017.

[7] Arora D, Rada C. A gendered model of the peasant household: Time poverty and farm production in rural Mozambique, Feminist Economics. 2017;23(2):93–119. 10.1080/13545701.2016.1220676.

[8] Diener E, Emmons RA, Larsen RJ, Griffin S (1985). The satisfaction with life scale. J Pers Assess.

[9] Pavot W, Diener E, Suh E (1998). The temporal satisfaction with life scale. J Pers Assess.

[10] IDMC (2018). Global Report on Internal Displacement.

[11] Norwegian Refugee Council Off the Agenda: The need to refocus the world’s attention on internal displacement, Briefing Paper 2017, Norway, www.nrc.no. Accessed 03 June 2020.

[12] Verwimp P, Maystadt J. Forced displacement and refugees in Sub-Saharan Africa: An Economic enquiry. 2015. World Bank Group African Region, Policy Research Working Paper 7517.World Bank, Washington, DC. https://openknowledge.worldbank.org/handle/10986/23481.

[13] Drakulic AM (2012). A phenomenological perspective on subjective well-being: from myth to science. Psychiatr Danub.

[14] Sirgy MJ. The psychology of quality of life: hedonic well-being, life satisfaction, and eudaimonia, 2^nd^ edition. 2012; social indicators research series Vol. 50, Netherlands, Springer. 10.1007/978-94-007-4405-9.

[15] IDMC. The Many Faces of Displacement: IDPs in Zimbabwe. 2008. www.internal-displacement.org. Accessed 17 July 2012.

[16] United Nations Guiding Principles on Internal Displacement. U. N Office of the Commissioner for Human Rights. 2004. https://www.unhcr.org/protection/idps/43ce1cff2/guiding-principles-internal-displacement.html. Acessed 10 June 2021.

[17] Opukri CO, Ibaba IS (2008). Oil induced environmental degradation and internal population displacement in the Nigeria’s Niger Delta. Justainable Dev Afr.

